# A Q-learning-based routing scheme for smart air quality monitoring system using flying ad hoc networks

**DOI:** 10.1038/s41598-022-20353-x

**Published:** 2022-11-23

**Authors:** Jan Lansky, Amir Masoud Rahmani, Seid Miad Zandavi, Vera Chung, Efat Yousefpoor, Mohammad Sadegh Yousefpoor, Faheem Khan, Mehdi Hosseinzadeh

**Affiliations:** 1 Department of Computer Science and Mathematics, Faculty of Economic Studies, University of Finance and Administration, Prague, Czech Republic; 2 Future Technology Research Center, National Yunlin University of Science and Technology, Yunlin, Taiwan; 3 School of Biotechnology and Biomolecular Science, The University of New South Wales, Sydney, Australia; 4 School of Computer Science, The University of Sydney, Sydney, Australia; 5 Department of Computer Engineering, Dezful Branch, Islamic Azad University, Dezful, Iran; 6 Department of Computer Engineering, Gachon University, Seongnam, Republic of Korea; 7 Institute of Research and Development, Duy Tan University, Da Nang, Vietnam; 8 School of Medicine and Pharmacy, Duy Tan University, Da Nang, Vietnam; 9 Department of Computer Science, University of Human Development, Sulaymaniyah, Iraq

**Keywords:** Environmental sciences, Engineering, Mathematics and computing

## Abstract

Air pollution has changed ecosystem and atmosphere. It is dangerous for environment, human health, and other living creatures. This contamination is due to various industrial and chemical pollutants, which reduce air, water, and soil quality. Therefore, air quality monitoring is essential. Flying ad hoc networks (FANETs) are an effective solution for intelligent air quality monitoring and evaluation. A FANET-based air quality monitoring system uses unmanned aerial vehicles (UAVs) to measure air pollutants. Therefore, these systems have particular features, such as the movement of UAVs in three-dimensional area, high dynamism, quick topological changes, constrained resources, and low density of UAVs in the network. Therefore, the routing issue is a fundamental challenge in these systems. In this paper, we introduce a Q-learning-based routing method called QFAN for intelligent air quality monitoring systems. The proposed method consists of two parts: route discovery and route maintenance. In the part one, a Q-learning-based route discovery mechanism is designed. Also, we propose a filtering parameter to filter some UAVs in the network and restrict the search space. In the route maintenance phase, QFAN seeks to detect and correct the paths near to breakdown. Moreover, QFAN can quickly identify and replace the failed paths. Finally, QFAN is simulated using NS2 to assess its performance. The simulation results show that QFAN surpasses other routing approaches with regard to end-to-end delay, packet delivery ratio, energy consumption, and network lifetime. However, communication overhead has been increased slightly in QFAN.

## Introduction

Industrial growth has led to technological advances in the world, but these advances have forced industrial and chemical contaminations that negatively affect human health and environment, including agricultural products, insects, and animals^1,2^. Air quality standards are usually evaluated by measuring various air pollutants, including nitrogen dioxide ($NO_{2}$), ground-level ozone ($O_{3}$), particulate matter (PM), carbon dioxide ($CO_{2}$), and carbon monoxide (*CO*). These pollutants cause serious health injuries. In the long-term, they damage the human respiratory system and lead to his early death^2,3^. For this reason, air quality monitoring is very important and vital. This issue has been considered by environmental organizations and governmental institutions. Traditional air quality monitoring systems are dependent on fixed monitoring stations. Although, these solutions are faced many challenges and restrictions because these stations can cover a limited area, and their deployment is difficult and costly^4–6^. Today, air quality in urban, rural, and industrial areas is controlled using several advanced networking technologies such as flying ad hoc networks (FANETs), Internet of things (IoT), vehicular ad hoc networks (VANETs), and wireless sensor networks (WSNs)^7,8^.

**Figure 1:**
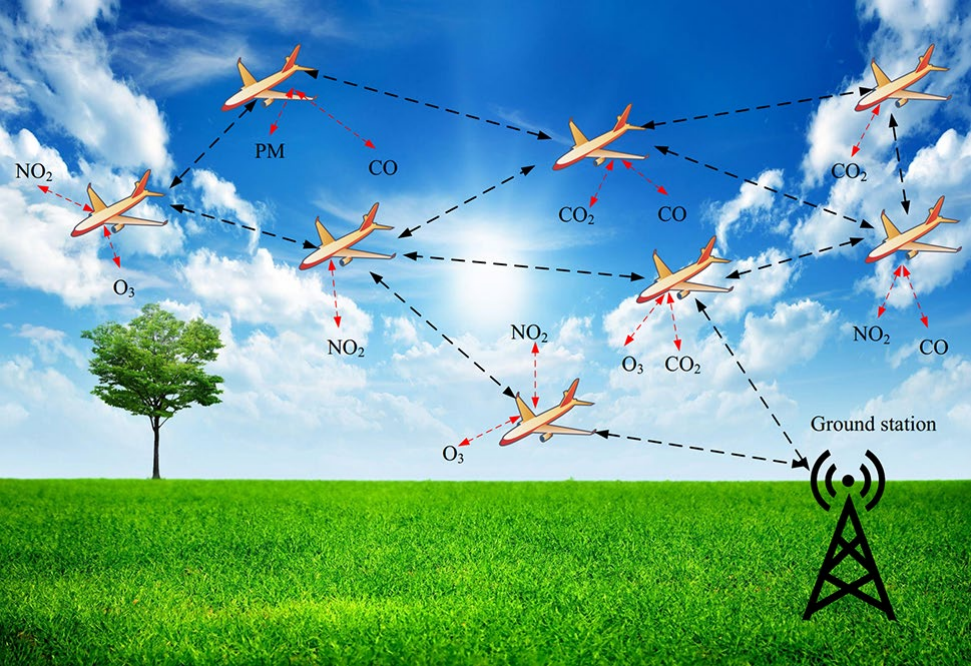
FANET for air quality monitoring.

Among these technologies, FANETs have special importance because these networks can cover a large operational environment and have been deployed easily and with acceptable costs^9,10^. FANET includes a group of unmanned aerial vehicles (UAVs) organized in an ad hoc form. UAVs, which are also called drones, are known as a promising technology for intelligent air quality monitoring. UAVs move in a three-dimensional space, and their speed is usually in the range of 30–46 km/h. UAVs have powerful computing power and energy resources because they require a lot of energy to fly, which is much more compared to the energy needed for data processing^10,11^. Although, energy is a serious issue for small drones. Figure 1 shows the FANET application for smart air quality monitoring. Today, some researchers study the application of UAVs for intelligent air pollution monitoring systems^12^. For example, in^13^, the authors have presented a component for the UAV structure to control air pollutants using a real-time manner. In^14^, UAVs and mobile stations are combined with each other to automatically monitor air pollution parameters around the roads. In^15^, the researchers have presented experiments to evaluate water quality using UAVs in southwest China. In^16^, the authors have expressed the importance of UAVs to monitor hazardous pollutants such as leakage of chemical materials. Furthermore, in^17^, UAVs and IoT have been integrated with each other to monitor air pollution parameters. In FANET-based air quality monitoring system, each UAV is equipped with cameras and specific sensors to measure air pollutants. With this equipment, UAVs can collect their environmental data and send them to ground station (GS) using wireless communication technologies.

Flying ad hoc networks have special features such as low density, rapid topological changes, quick changes in UAV conditions such as removing and adding UAVs. These features cause link failure when sending data related to the measurement of air pollutants to the ground station (GS). In an intelligent air quality monitoring system based on FANET, the time required to deliver data packets from source to destination should be short, and collision between packets should not occur. Due to the specific features of FANETs, providing an efficient routing method for this smart system is challenging^18^. Routing means sending and receiving packets between two UAVs called source UAV and destination UAV. An appropriate routing process helps the intelligent air quality monitoring system to stably and continuously control air quality in an environment. In different studies, researchers have stated various routing methods for flying ad hoc networks, for example position-based, topology-based, hybrid, and nature-inspired routing methods^18–20^. Existing classical routing protocols cannot provide a good solution to meet the routing problem in FANET. Recently, many researchers have tried to solve the routing challenge in FANETs using artificial intelligence (AI) and machine learning (ML) techniques^21^. Therefore, UAVs can independently learn the routing process in FANET. In ML, reinforcement learning (RL) is an efficient technique, which can efficiently and independently design routing algorithms in FANETs. RL is popular due to the use of trial and error technique. This algorithm includes an agent and an environment^22^. In a reinforcement learning-based routing method, the agent discovers the network environment by performing appropriate actions to achieve an optimal routing policy. To this end, the agent must discover the best paths between different source-destination pairs by considering the optimization criteria. Usually, this process uses the local information of nodes to lower energy consumption and improve network connections. Although to achieve an optimal routing technique, it is necessary to identify the total system^23,24^.

In this paper, we propose a Q-learning-based routing method called QFAN for the intelligent air quality monitoring system using FANETs. QFAN includes two parts: route discovery and route maintenance.

- **Route discovery:** QFAN uses a Q-Learning-based route discovery mechanism to discover a new path between source-destination pairs. In this mechanism, the route request packet (RREQ) plays the role of an agent. Furthermore, the network is the learning environment. The state of the agent indicates the node, which has received the RREQ message. In this process, the action represents a set of allowed neighboring nodes, which can receive the RREQ message in the current state. In the route discovery process, we present the filtering parameter according to several factors such as the motion direction, remaining energy, link quality, and distance to restrict the search space. As a result, the agent can achieve the optimal response (i.e. the best route) at a lower time. Additionally, QFAN utilizes a reward function, which considers three parameters, including delay, the number of hops, and route fitness.
- **Route maintenance:** It includes two parts. In the first part, QFAN detects and corrects routes close to failure. This helps QFAN to keep the data transmission process without interruption. In the second part, QFAN quickly identifies the failed paths to replace them with alternative paths to lower latency in the data transfer process.

In the following, the paper is organized as follows: in “Related works”, some routing approaches are reviewed in FANETs. “Basic concepts” introduces reinforcement learning, which is used for designing QFAN. Also, the system model used in our proposed approach is presented in this section. “Proposed method” describes QFAN in detail. “Simulation and evaluation of results” provides the simulation results of our scheme. Finally, the paper is concluded in “Conclusion”.

## Related works

Arafat and Moh in^25^ have presented the Q-learning-based topology-aware routing (QTAR) scheme for FANETs. To create reliable paths, QTAR not only utilizes the information of single-hop neighbors but also uses the information of two-hop neighbors. The authors believe that this method improves the path discovery process, reduces the time required for calculating routes and improves the selection of the next-hop node. Although this technique increases routing overhead and system complexity. QTAR considers scales such as spatial information, delay, speed, and energy when selecting next-hop node. Moreover, QTAR provides a technique for calculating the link lifetime to estimate the Hello interval and the link holding time. In this protocol, Q-Learning factors such as learning rate and reward factor are adaptively determined with regard to network conditions. QTAR is executed on a 3D environment that is acceptable for FANET.

Jung et al. in^26^ have suggested the Q-Learning-based Geographic routing scheme (QGeo). It is a distributed scheme, which uses local information for routing decisions. This method seeks to reduce routing overhead and delay in the routing process. In this process, QGeo has used spatial information and node speed in the network and ignored parameters such as energy and delay. This issue may create low-energy, unstable, and high-delay routes. In this method, the discount factor in Q-learning is specified based on distance and the mobility pattern of neighbors. However, this approach is executed on two-dimensional space that is not compatible with FANET.

Karp and Kung in^27^ have provided the greedy perimeter stateless routing (GPSR) scheme for ad hac networks. GPSR applies the location information of the single-hop neighboring UAVs in routing decisions. GPSR diminishes routing overhead and accelerates the path discovery operation. This method uses a greedy routing technique to acquire the next-hop node. However, GPSR does not pay attention to parameters such as energy, delay, and speed of nodes in dynamic environments. As a result, it have limitations in the route selection process in FANETs.

Lee et al. in^28^ have offered the energy-aware fuzzy logic-based routing method for FANETs. This method, which is an improved model of AODV, builds stable routes in the network, distributes the energy consumption evenly between network nodes and increases the network lifetime. This routing method consists of two parts: finding and maintaining routing paths. In the first part, each node obtains a score to decide on the RREQ rebroadcasting. Only high-score nodes are tasked to disseminate RREQs in the network. Furthermore, this work manages the flood of RREQs in the network and prevents the broadcast storm problem. When choosing the path, a fuzzy system is designed to pick out paths with low delay, high fitness and less hops. The second part involves the prevention of the route failure via discovering and modifying the paths on the failure threshold and the reconstruction of failed paths via identifying and replacing rapidly these paths. This method is executed on the 3D environment that is acceptable for FANET. Although this approach has a lot of communication overhead.

Liu et al. in^29^ have suggested the Q-learning-based multi-objective optimization routing (QMR) in FANETs. QMR has improved the exploration and exploitation system to earn the most effective routes, shorten latency, and lessen energy consumption in the network. In QMR, nodes regularly disseminate hello messages to obtain information about neighboring UAVs to reliably select the next-hop node. In QMR, Q-learning factors (i.e. the learning rate and the discount factor) are adjusted according to network conditions. This issue helps QMR to better be adapted to the dynamic network. When these parameters are adjustable, the routing process is more accurate. This decreases disconnections in the network. QMR proposes a mechanism for preventing path failure. Although, it is executed on a two-dimensional area that is not matchable for FANET. Additionally, QMR does not design an appropriate control mechanism for controlling network congestion.

Oubbati et al. in^30^ have presented a routing technique called ECaD for FANETs. It is rooted in AODV. In ECaD, high-energy UAVs can participate in route discovery. This helps ECaD to create stable paths. However, this method is challenging when a node cannot find high-energy neighboring nodes as the next-hop node. Thus, this node cannot create any route to other nodes in the network. Therefore, this UAV is isolated in the network. In addition, ECaD considers only energy parameter when finding the next-hop node. This scheme evenly distributes energy consumption and builds stale paths in the network. Furthermore, it prevents link failure using a predictive mechanism. ECaD is simulated in a three-dimensional space.

Costa et al. in^31^ have proposed a Q-learning-based routing protocol called Q-FANET for flying ad hoc networks. Q-FANET can lower delay in the network. It has been inspired by two methods, including QMR and Q-Noise+. It includes two main parts: discovering neighbors and routing. In the neighbor discovery process, UAVs exchange their information periodically. This helps UAVs to be aware of their neighbors at any moment and use the information in the routing phase. In the second part, Q-FANET utilizes an improved Q-learning technique called Q-Learning+ to improve QMR and Q-Noise+. Q-FANET considers channel quality conditions when determining Q-values so that high-quality links can obtain a higher Q-value. Although this protocol does not pay attention to energy when finding routing paths. This approach is executed on a two-dimensional area that is not accepted for FANETs.

Perkins et al. in^32^ have offered the ad hoc on-demand distance vector (AODV) approach in ad hoc networks. This method is on-demand. This means that routing paths are constructed only when there is a route request. In recent years, many routing techniques are inspired by AODV. However, this method has very challenges in FANETs because of their properties such as high-speed flying nodes and fast link failure. Additionally, AODV has a long delay when discovering a new route. AODV introduces a maintenance mechanism to discover and correct the failed paths. AODV requires high bandwidth and high delay for creating paths and correcting the failed routes, especially in large-scale networks.

Rahmani et al. in^33^ have presented an enhanced model of the optimized link state routing protocol (OLSR) called OLSR+ for FANETs. They have suggested a novel solution for assessing the link lifetime by considering various scales, namely connection quality, distance, relative speed, and motion direction of flying nodes. In addition, OLSR+ has designed a fuzzy logic-based algorithm to choose multipoint relays (MPRs) in the network. This fuzzy model involves three fuzzy inputs: the remaining energy of UAV, the connection lifetime of UAVs, and their neighborhood degree. It allocates a score to each UAV. This score is used to select MPRs. In this method, the routing table is calculated by considering three parameters: the number of hops, path energy, and path lifetime. OLSR+ creates stable and energy-efficient paths and reduces packet loss. Moreover, this method is executed on a 3D space that is acceptable for FANET. However, OLSR+ suffers from a lot of routing overhead. Due to dynamic topology of FANETs, it is very difficult to update neighborhood table and routing table because it requires a lot of delay and communication overhead.

Qiu et al. in^34^ have designed a Q-learning-based geographic routing (QLGR) method for FANETs. This protocol utilizes a multi-agent learning algorithm for designing the routing model. In this model, each drone is regarded as an agent, and the whole network is assumed to be the learning system. The state set involves the status of nodes, and the action set indicates neighbors of the current UAV. In QLGR, each UAV uses a distributed technique to estimate the fitness value of its neighbors based on local information, namely link quality, energy, and queue length to choose the most suitable relay UAV. This information is obtained by broadcasting hello messages between the UAVs periodically. After each action, the agent receives two rewards from the environment: the local reward (LR) that represents the local returns of neighboring nodes and is calculated based on connection quality and load capacity, and the global reward (GR) that indicates global returns of UAVs after taking this action. GR is evaluated based on distance from the current location of the packet to the destination. This method presents a rebroadcast control mechanism to determine the broadcast interval of hello messages and manage the communication overhead in the network. This protocol is executed on a two-dimensional space that is not appropriate for FANET.

Table 1 expresses the most important strengths and weaknesses of the related works and compares these schemes with our scheme.

**Table 1:** Strengths and weaknesses of related works.

Method	Strengths	Weaknesses
QTAR^25^	Considering parameters such as energy, spatial information, delay, and movement of UAVs for discovering routes, adjusting the broadcast hello interval based on the path lifetime, adjusting learning rate and discount factor dynamically, implementing this routing method in a 3D space	High overhead because of the need to information of single-hop and two-hop neighbors
QGeo^26^	Adjusting the discount parameter dynamically by calculating the distance and movement pattern of UAVs	Not considering the residual energy of flying nodes and delay in the routing process, the possibility of making unstable and low-energy paths, implementing QGeo in a two-dimensional environment, enlarging Q-table by growing the number of nodes in the network
GPSR^27^	Reducing routing overhead and delay when creating paths, high scalability	Constructing paths only based on distance and ignoring parameters such as energy, delay, link quality in this process, the possibility of making unstable and low-energy routes, not suitable for 3D environments such as FANETs
lee et al.^28^	Distributing energy consumption uniformly in the network, prolonging network lifetime, building high-energy routes, designing a flood control mechanism of route request messages (RREQs) in the network, predicting route failure, simulating this routing approach in a 3D environment	Long delay when finding paths, high routing overhead
QMR^29^	Adjusting Q-Learning parameters adaptively, designing an adaptive mechanism to build a trade-off between exploration and exploitation, presenting a penalty mechanism for addressing the routing hole problem	Not designing a broadcast control mechanism, not considering energy parameter in the routing process, the possibility of forming unstable routes, simulating QMR in a two-dimensional environment
ECaD^30^	Considering the energy of UAVs when selecting paths, not participating low-energy nodes in the route construction operation, implementing the routing method in a 3D space	Flooding routing messages
Q-FANET^31^	Improving delay in the routing process, Predicting path failure	Implementing Q-FANET in a 2D space, not considering the energy parameter in the routing process, the possibility of forming unstable routes in the network, fixed Q-Learning parameters
AODV^32^	Designing an on-demand algorithm, designing a route maintenance model	High delay and routing overhead when creating a route, the possibility of the broadcast storm, not considering the energy parameter when finding paths, not considering the features of FANETs
OLSR+^33^	Implementing this routing method in a 3D space, calculating link lifetime based on connection quality, distance, relative speed, and motion direction of UAVs, presenting a fuzzy logic-based algorithm for selecting MPRs, considering the route energy and the path delay when choosing a route, reducing packet loss, improving stability of paths	high delay and routing overhead when updating routing table
QLGR^34^	Presenting a multi-agent routing method, improving the convergence speed of the learning algorithm, high scalability, providing a broadcast control technique of hello messages, reducing routing overhead	Difficulty in coordinating different agents in the learning process, high computational complexity, implementing this routing method in a two-dimensional space
QFAN	Implementing our scheme in a 3D space, filtering some states in the state space, limiting search space, and increasing the convergence speed of the proposed method, reducing delay when creating paths, improving scalability, considering the energy parameter and the movement pattern of UAVs in the routing operation, planing a mechanism to prevent route failure, rebuilding failed paths, improving network lifetime, reducing packet loss	High routing overhead and fixed Q-learning algorithm parameters

## Basic concepts

In this section, we demonstrate Q-leaning and system model briefly because we consider these concepts for designing the route discovery mechanism in QFAN.

### Reinforcement learning

**Figure 2:**
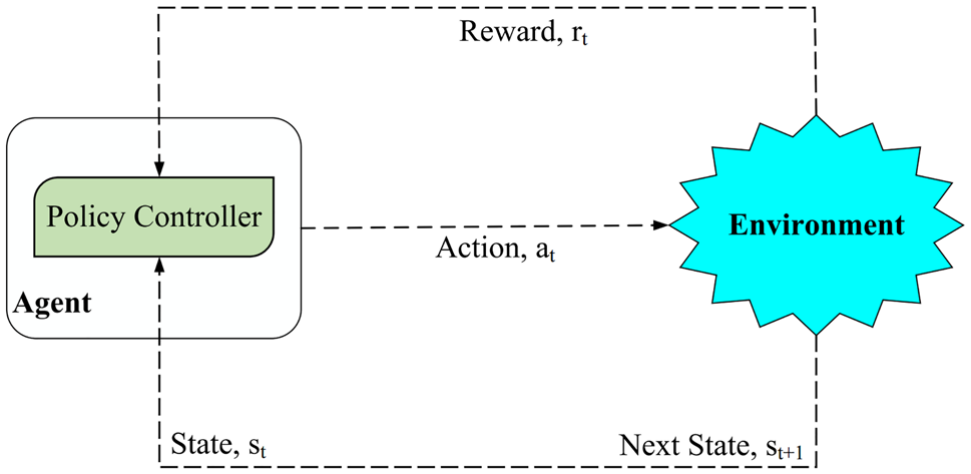
Reinforcement learning process.

Reinforcement learning (RL) is a robust and useful implement in artificial intelligence (AI). RL includes two main modules: agent and environment. In this approach, the agent takes various actions to explore the environment^35^. It decides on an action according to the Markov decision process (MDP) that is a useful structure for simulating various issues using dynamic programming and RL techniques. It helps the agent to control this process randomly. Usually, MDP is known based on four parameters that are displayed as $\left( S,A,p,r\right)$. So that *S* and *A* indicate the finite state and action sets, respectively. Moreover, *p* defines a probability value for replacing the current state with the next state after running the action *a*. Furthermore, *r* is a reward given by the environment after completing the action *a*. According to Fig. 2, at the time *t*, the agent takes the action $a_{t}$ with regard to its latest state $s_{t}$ to get the reward $r_{t}$ and its new state $s_{t+1}$ from the environment. The agent seeks to learn the policy $\pi$ that enlarges the reward obtained from the environment. In the long term, the expected discounted total reward must be increased through $\max \left[ \sum \nolimits _{t=0}^{T}{\delta {r_{t}}\left( {s_{t}},\pi \left( {s_{t}} \right) \right) } \right]$. $\delta \in \left[ 0,1 \right]$ is known as the discount factor, which indicates the importance of the reward and the effort of the agent for discovering the environment. In the proposed method, $\delta =0.7$. According to the discounted reward, when the transition probabilities (i.e. *p*) are known, the Bellman equation called Q-function (Eq. 1) is created to perform the next action $a_{t+1}$ using MDP.

1 $$\begin{aligned} Q\left( {s_{t}},{a_{t}}\right) =\left( 1-\alpha \right) Q\left( {s_{t}},{a_{t}}\right) +\alpha \left[ r+\delta \left( \max Q\left( {s_{t+1}},{a_{t}}\right) \right) \right] , \end{aligned}$$

where $\alpha$ represents the learning rate so that $0<\alpha \le 1$. $\alpha$ (also called step size) is one of the Q-learning parameters. It is between 0 and 1. If $\alpha = 0$, then the Q-values are never updated. As a result, the agent does not learn any new knowledge and only exploits prior knowledge, else if $\alpha = 1$, this means that the learning process is very fast and the agent focuses only on the most recent information and ignores its prior knowledge when exploring possibilities. In practice, researchers usually consider a constant learning rate, such as $\alpha = 0.1$^36^. In the proposed method, $\alpha =0.1$.

The Q-Learning algorithm presents a model-free and off-policy RL technique, which uses Q-function presented in Equation 1. It helps an agent to learn the best actions^35,37^. Q-learning applies a table (i.e. Q-Table) for storing all optimal state-action pairs. In this table, there are two inputs (i.e. the state-action pairs) and one output called Q-value. Q-Learning seeks to achieve maximum Q-value. To this end, agents learn the best action strategy through the environmental feedback. According to the learning operation, the agent analyzes whether an action is appropriate in the current state or not. Based on this decision, the agent chooses a better action in the next step. Q-values are refreshed in each step according to Eq. (1). Then, the agent exploits the environment by performing actions with most suitable Q-value. This policy is known as $\epsilon$-greedy. According to this policy, the agent explores or exploits the environment based on the probability value $\epsilon$.

### System model

**Figure 3:**
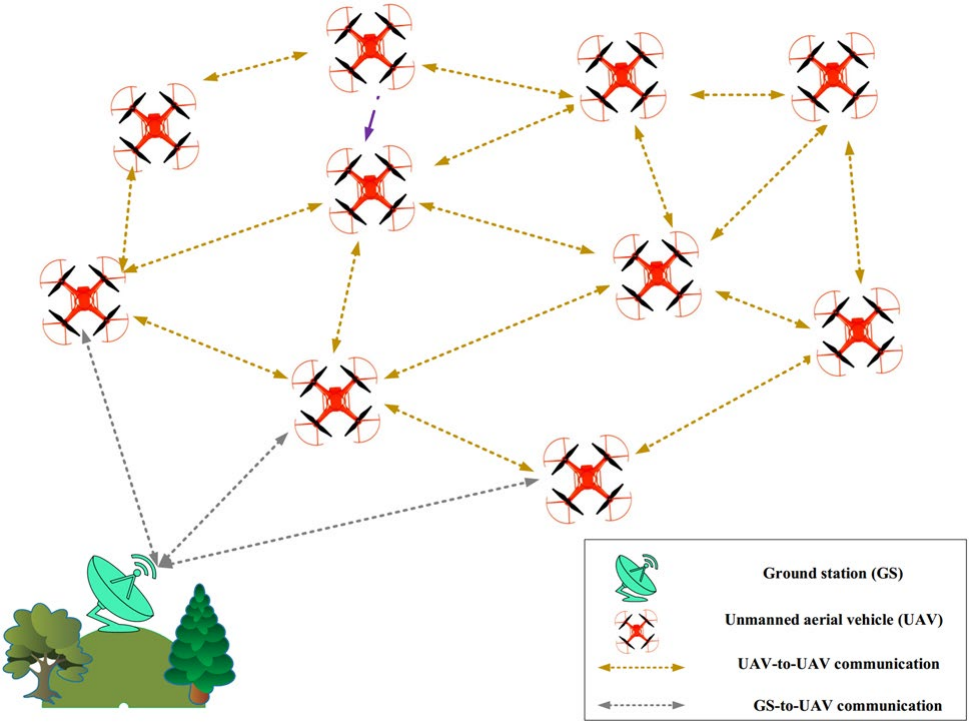
Network model in QFAN.

This section illustrates the network system applied in QFAN. This network model is homogeneous and consists of a number of flying nodes. They are randomly scattered in the network and fly in a three-dimensional space. These drones includes similar energy source, computing power, and storage capacity. In each UAV, the wireless interface in the Mac layer includes IEEE 802.11n that has suitable features such as high throughput, high data rate, support from long-range communication, high resistance to interference, and uniform coverage within an area^38^. The flying nodes move very fast, and distance between them varies at any moment. Each UAV consists of a special ID. They are equipped with a localization mechanism like global positioning system (GPS). Therefore, UAVs know their position and speed at any moment. In QFAN, the network model utilizes two connection types, namely UAV-to-UAV and UAV-to-GS. The system model is illustrated in Fig. 3.

## Proposed method

In this section, we present a Q-Learning routing approach called QFAN in FANETs. QFAN improves AODV protocol using Q-learning algorithm. Note that AODV is a well-known reactive algorithms defined in ad hoc networks because of its main property (i.e. it is on-demand). Today, many algorithms such as^28^ are inspired by AODV because it is valuable and useful. However, AODV cannot be applied in FANETs because it does not consider the unique properties of these networks, for example high-mobile UAVs, and frequent disconnections. Therefore, we try to improve this routing protocol to be suitable for FANETs. In QFAN, we consider different parameters such as link quality, motion direction, route delay, distance between nodes, and energy to create stable paths with low delay and rise the packet delivery rate (PDR). QFAN seeks to extend the network lifetime by distributing the consumed energy between network nodes uniformly. QFAN involves two parts: 1) Route discovery 2) Route maintenance. In the following, we illustrate each part item by item.

### Route discovery

In QFAN, when a source node ($U_{S}$) wants to build a connection link between itself and the destination node ($U_{D}$) for exchanging data packets, $U_{S}$ examines its routing table for achieving a reliable path. If $U_{S}$ cannot obtain a valid path between itself and $U_{D}$, it uses a Q-Learning-based routing operation to discover the best path ($Best_{Route}$) for exchanging data packets. In this process, each flying node ($U_{i}$) exchanges Hello messages with its neighboring UAVs at regular time intervals. This message contains spatial information $\left( x_{i}^{t},y_{i}^{t},z_{i}^{t}\right)$, velocity vector $\left( v_{x,i}^{t},v_{y,i}^{t},v_{z,i}^{t}\right)$, delay information, Q-value, and energy ($E_{i}^{t}$) at the time *t*. $U_{i}$ stores information about its neighbors in a neighboring table. Based on information stored in this table, $U_{i}$ calculates the filtering parameter ($S_{filtering}$) for each neighboring node such as $U_{j}$. This parameter helps $U_{i}$ to filter a number of its neighbors that are not in good conditions for rebroadcasting RREQ, and put other neighbors in a set of licensed neighbors ($Set_{Licensed}$). For calculating $S_{filtering}$, $U_{i}$ uses four parameters, including motion direction, remaining energy, link quality, and distance.

#### Motion direction ($\theta _{i,j}^{t}$)

$U_{i}$ calculates the angle between itself and $U_{j}$ at the time *t*. If the movement direction of two nodes $U_{i}$ and $U_{j}$ is similar (i.e. the angle between $U_{i}$ and $U_{j}$ is zero), $U_{i}$ increases $S_{filtering}$ corresponding to $U_{j}$ to achieve more chance to be placed in $Set_{Licensed}$ because the communication link between $U_{i}$ and $U_{j}$ is valid for a longer time. Thus, more stable paths are created. Equation (2) calculates the angle between the motion directions of $U_{i}$ and $U_{j}$.

2 $$\begin{aligned} \theta _{i,j}^{t}={{\cos }^{-1}}\left( \frac{v_{x,j}^{t}v_{x,i}^{t}+v_{y,j}^{t}v_{y,i}^{t}+v_{z,j}^{t}v_{z,i}^{t}}{\left| \mathbf {V}_{i}^{t} \right| \left| \mathbf {V}_{j}^{t} \right| } \right) ,\quad 0\le \theta \le \pi , \end{aligned}$$

where $\left( v_{x,i}^{t},v_{y,i}^{t},v_{z,i}^{t}\right)$ and $\left( v_{x,j}^{t},v_{y,j}^{t},v_{z,j}^{t}\right)$ are velocity vectors of $U_{i}$ and $U_{j}$, respectively. In addition, $\left| \mathbf {V}_{i}^{t}\right|$ and $\left| \mathbf {V}_{i}^{t}\right|$ indicate the length of these vectors. They are obtained through Eqs. (3) and (4), respectively.

3 $$\begin{aligned} \left| \mathbf {V}_{i}^{t}\right| =\sqrt{{{\left( v_{x,i}^{t}\right) }^{2}}+{{\left( v_{y.i}^{t} \right) }^{2}}+{{\left( v_{z,i}^{t}\right) }^{2}}}. \end{aligned}$$

Also,

4 $$\begin{aligned} \left| \mathbf {V}_{j}^{t}\right| =\sqrt{{{\left( v_{x,j}^{t}\right) }^{2}}+{{\left( v_{y.j}^{t} \right) }^{2}}+{{\left( v_{z,j}^{t}\right) }^{2}}}. \end{aligned}$$

#### Remaining energy ($E_{j}^{t}$)

$U_{i}$ increases $S_{filtering}$ corresponding to high-energy neighboring nodes to achieve more chance for being placed in $Set_{Licensed}$. The purpose of choosing $E_{j}^{t}$ is to distribute energy consumption uniformly in the network. According to this parameter, high-energy nodes are responsible for discovering new paths and forming stable paths.

#### Link quality ($Q_{i,j}^{t}$)

$U_{i}$ increases $S_{filtering}$ corresponding neighbors that include a better connection quality compared to others. This increases their chances for being placed in $Set_{Licensed}$. The purpose of choosing $Q_{i,j}^{t}$ is to construct high-quality paths. When there is a low-quality link between $U_{i}$ and $U_{j}$, the path is unstable. This causes path failure. In QFAN, we assume that the link quality is evaluated using the received signal strength indication (RSSI). It can exactly assess link quality because research literature show that the higher RSSI leads to a better packet delivery rate (PDR) in the receptor and the transmitter^39,40^. Also, RSSI is stable for a short time period (about 2 s) and its standard deviation is close to 1 dBm^41^. In our scheme, we assume that RSSI registers are embedded in radio transceivers to calculate the signal strength when receiving packets.

#### Distance ($D_{i,j}^{t}$)

If the distance between $U_{i}$ and its neighbor such as $U_{j}$ is suitable, $U_{i}$ increases $S_{filtering}$ corresponding to $U_{j}$. Thus, $U_{j}$ achieves more chance for being in $Set_{Licensed}$. The suitable distance means that $U_{i}$ and $U_{j}$ are not very close to each other because if $U_{j}$ is selected as relay node, it rises the hop count in the path, and this issue does not help $U_{i}$ to find the path. On the other hand, the suitable distance means that $U_{i}$ and $U_{j}$ are not very far away from each other because these two nodes quickly get out from each other communication range. Therefore, the path created by these nodes will be invalid quickly. The suitable distance between the two nodes is called $D_{i,j}^{t}$ and is between ${D_{Min}}$ and ${D_{Max}}$, where $0\le {D_{Min}}<{D_{Max}}$ and $0<{D_{Max}}\le R$. *R* indicates the transmission radius of UAVs in the network. In this case, $U_{i}$ and $U_{j}$ can form stable paths. $D_{i,j}^{t}$ is the Euclidian distance between $U_{i}$ and $U_{j}$ that is computed through Eq. (5):

5 $$\begin{aligned} D_{i,j}^{t}=\left\{ \begin{array}{ll} 1-\frac{\left| \sqrt{{{\left( x_{i}^{t}-x_{j}^{t} \right) }^{2}}+{{\left( y_{i}^{t}-y_{j}^{t} \right) }^{2}}+{{\left( z_{i}^{t}-z_{j}^{t} \right) }^{2}}} \right| }{{D_{Min}}},&{}\quad 0\le D_{i,j}^{t}<{{D}_{Min}} \\ 1,&{}\quad {D_{Min}}\le D_{i,j}^{t}\le {D_{Max}} \\ 1-\frac{\left| \sqrt{{{\left( x_{i}^{t}-x_{j}^{t} \right) }^{2}} +{{\left( y_{i}^{t}-y_{j}^{t} \right) }^{2}}+{{\left( z_{i}^{t} -z_{j}^{t} \right) }^{2}}}-{D_{Max}} \right| }{R-{D_{Max}}}, &{}\quad {D_{Max}}<D_{i,j}^{t}\le R \\ \end{array} \right. , \end{aligned}$$

where $\left( x_{i}^{t},y_{i}^{t},z_{i}^{t}\right)$ and $\left( x_{j}^{t},y_{j}^{t},z_{j}^{t}\right)$ indicate the spatial coordinates of $U_{i}$ and $U_{j}$, respectively.

After calculating these parameters, $U_{i}$ calculates $S_{filtering_{j}}$ corresponding to $U_{j}$ through Eq. (6):

6 $$\begin{aligned} {{S}_{filterin{{g}_{j}}}}={{\omega }_{1}}\left( 1-\frac{\theta _{i,j}^{t}}{\pi }\right) +{{\omega }_{2}}\left( \frac{E_{j}^{t}-{{E}_{\min }}}{{{E}_{\max }}-{{E}_{\min }}} \right) +{{\omega }_{3}}\left( \frac{Q_{i,j}^{t}-{{Q}_{\min }}}{{{Q}_{\max }}-{{Q}_{\min }}} \right) +{{\omega }_{4}}\left( D_{i,j}^{t} \right) , \end{aligned}$$

where ${E_{\min }}\ge 0$ indicates the minimum energy (20% of initial energy) and ${E_{\max }}>0$ is the primary energy of UAVs. In addition, ${Q_{\min }}\ge 0$ and ${Q_{\max }}>0$ are the least link quality and the highest link quality between two nodes, respectively. According to^39^, PDR is zero when $RSSI=0$. Also, PDR is about 99% and when $RSSI=-87\,\,dBm$. We consider these values as ${Q_{\min }}$ and ${Q_{\max }}$, respectively. ${{\omega }_{1}}$, ${{\omega }_{2}}$, ${{\omega }_{3}}$, and ${{\omega }_{4}}$ are weight coefficients, and ${{\omega }_{1}}+{{\omega }_{2}}+{{\omega }_{3}}+{{\omega }_{4}}=1$. These coefficients indicate the effect of each factor on $S_{filtering_{j}}$ in Eq. (6). The important point is that the sum of all weight coefficients must be one. This paper considers similar values for these coefficients, i.e. ${{\omega }_{1}}={{\omega }_{2}}={{\omega }_{3}}={{\omega }_{4}}=\frac{1}{4}$. Analysis of the effect of these coefficients is outside the scope of this paper.

Now, we have standardized $S_{filtering_{j}}$ using Eq. (7). It evaluates the number of standard deviations by which $S_{filtering_{j}}$ is above or below the mean value. $S_{filtering_{j}}$ above the mean has a positive value, while $S_{filtering_{j}}$ below the mean has a negative value. A standardized value is computed by subtracting the population mean from an individual raw score and then dividing the difference by the population standard deviation. For more details, refer to^42^.

7 $$\begin{aligned} S_{Standard_{j}}=\frac{{S_{filtering_{j}}}-\mu }{\sigma }, \end{aligned}$$

$\mu$ and $\sigma$ indicate the mean value and standard deviation of $S_{filtering}$ of neighbors, respectively:

8 $$\begin{aligned} \mu= & {} \frac{1}{{{n}_{neighbor}}}\sum \limits _{i=1}^{{ {n}_{neighbor}}}{{{S}_{filtering_{i}}}}. \end{aligned}$$

9 $$\begin{aligned} \sigma= & {} \sqrt{\frac{1}{{n_{neighbor}}}\sum \limits _{j=1}^{{n_{neighbor}}}{{{\left( {S_{filtering_{j}}}-\mu \right) }^{2}}}}, \end{aligned}$$

where $n_{neighbor}$ indicates the number of neighbors of $U_{i}$.

Now, if $S_{Standard_{j}}\ge 0$, $U_{j}$ is placed in $Set_{Licensed}$. In QFAN, each RREQ message plays the role of an agent. The RREQ format in QFAN is similar to that in the AODV protocol. However, it includes two additional fields: $F_{Route}$ and $T_{Route}$. $F_{Route}$ represents the route fitness, which is in $\left[ 0,1 \right]$. It will be equal to the least value of the filtering parameter of nodes that create a path. This parameter is calculated in accordance with Eq. (10):

10 $$\begin{aligned} {F_{Route_{i}}}=\underset{{U_{j}}\in Route_{i}}{{\min }}\,\left( {S_{filtering_{j}}} \right) ,\quad j=1,\ldots ,{M_{i}}, \end{aligned}$$

where $M_{i}$ is the number of $U_{j}$ in $Route_{i}$.

$T_{Route}$ indicates the route delay, and its value is obtained from the total time needed to forward RREQ from $U_{S}$ to $U_{D}$. This parameter is calculated using Eq. (11):

11 $$\begin{aligned} {T_{Route_{i}}}=\sum \limits _{i=1}^{{M_{i}}}{{t_{i,i+1}}}, \end{aligned}$$

where $M_{i}$ is the number of flying nodes in $Route_{i}$. Also, ${t_{i,i+1}}$ indicates the single-hop delay between two nodes, including $U_{i}$ and $U_{i+1}$. This is the time needed to forward RREQ from $U_{i}$ to $U_{i+1}$, we use the equation presented in^29^. According to^29^, ${t_{i,i+1}}$ is calculated by two parameters, including the medium access delay (${D_{mac_{i,i+1}}}$) and the queuing delay (${D_{que_{i,i+1}}}$). ${D_{mac_{i,i+1}}}$ is the time needed for the medium access protocol to deliver this message or delete the duplicated message. ${D_{que_{i,i+1}}}$ illustrates the time interval required for the message to reach the head of the transfer queue. Note that^29^ does not considers the propagation delay because this delay is very short and depends on the light speed. As a result, ${t_{i,i+1}}$ is expressed based on Eq. (12):

12 $$\begin{aligned} {t_{i,i+1}}={D_{que_{i,i+1}}}+{D_{mac_{i,i+1}}}. \end{aligned}$$

Note that our learning system regards the network as the environment. The state of agent represents the flying node (i.e. $U_{i}$), which has received the RREQ message. In this process, the action represents a set of licensed neighboring nodes (i.e. ${U_{neg_{i}}}\in Set_{Licensed}$), which can receive the RREQ message in the current state (i.e. $U_{i}$). This set is displayed as $A=\left\{ {U_{i}}\rightarrow {U_{neg_{1}}},{U_{i}}\rightarrow {U_{neg_{2}}},\ldots ,{U_{i}}\rightarrow {U_{neg_{k}}} \right\}$. When the agent (i.e. the RREQ message) is in the state $U_{i}$ and takes the action ${U_{i}}\rightarrow {U_{j}}$, the state is converted into ${U_{j}}$. When starting the route discovery operation, we initialize different Q-Learning factors, like the learning rate ($\alpha$), discount factor ($\gamma$) and Q-value.

In the following, we demonstrate how to obtain the reward function. It is a major component of Q-Learning. The reward function indicates environmental feedback relative to the action ${a_{t}}$ taken in the state ${U_{t}}$. The proposed learning model must maximize the reward value when transferring RREQ from $U_S$ to $U_D$. In QFAN, we consider three scales, including route time (${T_{Route}}$), the number of hops ($hop_{count}$), and the route fitness ($F_{Route}$), to design the reward function for discovering the best path ($Best_{Route}$) to transfer data packets from $U_{S}$ to $U_{D}$. Thus, $Best_{Route}$ is a route that has the highest fitness, the lowest hops, and the lowest delay.

- ${F_{Route}}$: When ${F_{Route}}$ is close to one, the discovered route consists of high-energy nodes and high-quality links. Moreover, the distance between these nodes is suitable and their movement direction is similar. This guarantees that the created path is stable for longer time.
- $hop_{count}$: When the number of hops is low in one path, that path has less delay when transferring data packets. It enhances the performance of QFAN.
- ${T_{Route}}$: It is an essential factor when finding various paths in FANETs because the high velocity of flying nodes causes link failure and unstable paths. When delay is high in a path, the paths may be disconnected before transferring data to $U_{D}$. This increases packet loss in the network.

Therefore, the reward function is calculated according to Eq. (13):

13 $$\begin{aligned} Reward\left( {U_{t}},{a_{t}} \right) =\left\{ \begin{array}{ll} {R_{\max }},&{}\quad {U_{t+1}}\,is\,\ destination. \\ {{R}_{\min }},&{}\quad {U_{t+1}}\,is\,\ local\,minimum. \\ \left( 1-\frac{{T_{Route}}}{\max \left( {T_{Route}} \right) } \right) +\left( 1-\frac{hop_{count}}{N-1} \right) +{F_{Route}},&{}\quad Otherwise \\ \end{array} \right. , \end{aligned}$$

where ${T_{Route}}$ indicates delay in the path from ${U_{S}}$ to ${U_{t+1}}$. $\max \left( {T_{Route}}\right)$ is the maximum delay in the discovered paths to ${U_{t+1}}$. Moreover, $hop_{count}$ indicates the number of hops in the path. This parameter is inserted in RREQ. *N* is the total number of UAVs. With regard to the reward function, if the next-hop node is $U_{D}$, the link between ${U_{t}}$ and ${U_{t+1}}$ acquires the most reward value. On the other hand, when a local minimum occurs, i.e. among the states that can be selected, those that are farther away from $U_{D}$ compared to the current node acquire the minimum reward. In other modes, the reward function is evaluated using the route delay, the number of hops and the route fitness.

In QFAN, the convergence condition is the time required to find the best path ($Best_{Route}$) by the learning algorithm. In our scheme, when the learning algorithm are repeated five steps and the response remains unchanged, this means that the algorithm has converged and achieved the optimal response ($Best_{Route}$). Then, ${U_{S}}$ records the novel path in its routing table. Algorithm 1 expresses the pseudo-code of this operation.
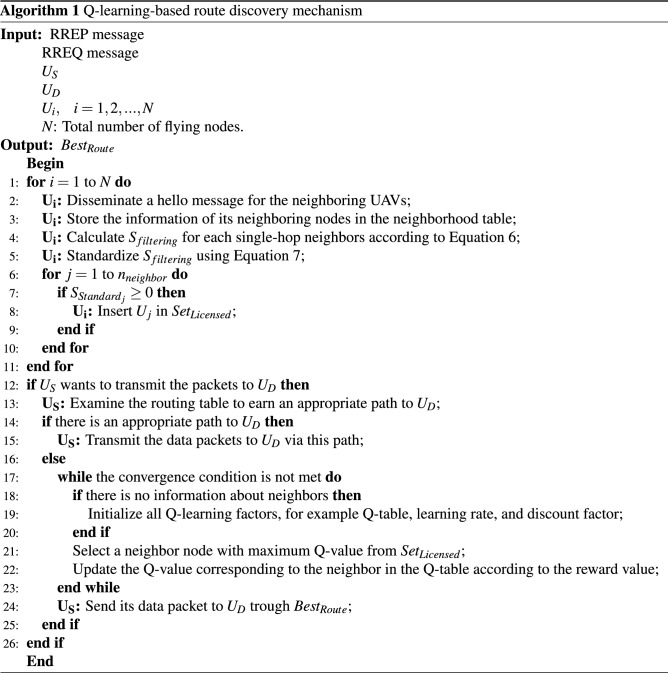


### Route maintenance

The existing paths may be disconnected because of the specific properties of FANETs, like the high-speed UAVs and the very dynamic topology. In the route maintenance process, the goal is to immediately detect a failed path and replace a new path for transferring data packets. When one of the following modes is met, the created path should be corrected to avoid its failure:

- **Mode 1:** When $U_{i}$ is dying in a path, meaning that its energy level is lower than the energy threshold $E_{Threshold}$ (i.e. $E_{U_{i}}<E_{Threshold}$). Thus, this path should be replaced because $U_{i}$ cannot continue the data transfer process.
- **Mode 2:** When the traffic level of $U_{i}$ in one path is more than the traffic threshold $Tr_{Threshold}$ (i.e. $Tr_{U_{i}}>Tr_{Threshold}$), its buffer capacity is at the overflow state. Therefore, the path has very high delay when transferring data because the path is blocked. Therefore, this path should be replaced because it cannot forward data packets.
- **Mode 3:** When the communication link between $U_{i}$ and $U_{j}$ is disconnecting in a path, meaning that its connection quality is lower than the quality threshold $Q_{Threshold}$ (i.e. ${Q_{Link_{i-j}}}<Q_{Threshold}$). Therefore, this path should be corrected.

If one of the three modes occurs, the reward corresponding to $U_{i}$ will be equal to $R_{\min }$. Then, $U_{i}$ searches its routing table and finds the paths that include itself. $U_{i}$ sends a feedback containing the reward value to its previous-hop nodes. Then, the previous-hop node executes the Q-Learning algorithm to pick out a new UAV as the next-hop node and modify the path. Note that the old route is valid and used for transferring data packet until the new path is discovered and replaced. After creating the new path, the old path is removed and data packets are sent through the new path.

To discover and replace failed paths, UAVs must regularly control the paths recorded in their routing table. To do this, $U_{S}$ regularly transmits the path control message to $U_{D}$ via the created path. If $U_{D}$ receives this message successfully, this path is still valid. Then, $U_{D}$ sends the ACK message to $U_{S}$. Otherwise, if $U_{D}$ does not receive the message on time, $U_{S}$ concludes that the path is cut off. At this step, the reward value related to the failed UAV will be equal to $R_{\min }$. This node sends this feedback to its previous-hop UAV to correct the failed path. Therefore, the previous-hop node runs the Q-Learning algorithm to specify a new UAV as the next-hop node and make a new path. Algorithm 2 presents the pseudo-code of the route maintenance operation.
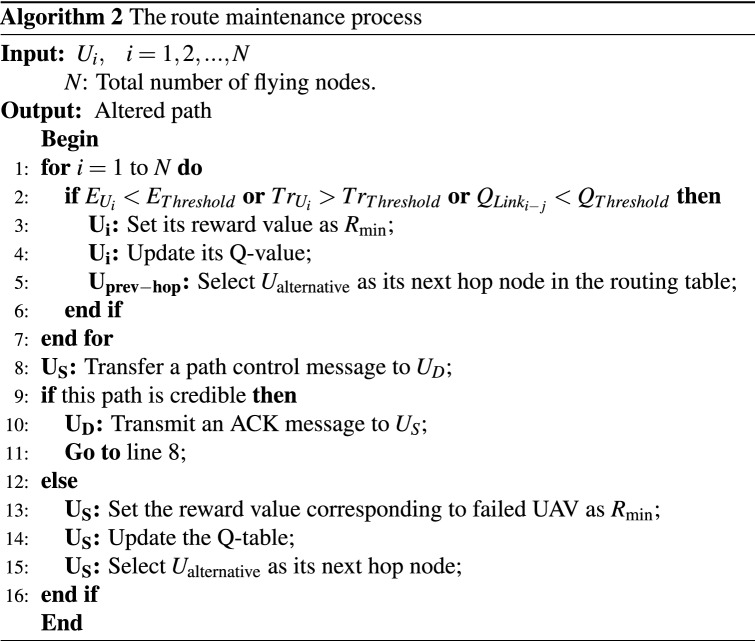


## Simulation and result evaluation

In this section, QFAN is implemented using the network simulator version 2 (NS2) to analyze its results. For this purpose, QFAN is compared with three routing schemes, namely QTAR^25^, GPSR^26^ and QGeo^27^ in terms of five parameters, including packet delivery rate (PDR), average end-to-end delay (Average E2E), communication overhead, consumed energy, and network lifetime. In the simulation process, the three-dimensional Gauss Markov mobility model (3D GM) simulates drone movement in the network environment^43^. The network dimensions are $2000\times 2000\times 300\,\,m^{3}$. Furthermore, the number of UAVs changes between 20 and 100, and their velocities are different from 10 m/s to $40\,$m/s. The initial energy of UAVs is $1000\,$J, and their communication radius is $250\,$m. In the simulation process, the IEEE 802.11n standard is regarded as a wireless interface in the MAC layer because this standard has suitable features such as high throughput, high data rate, support from long-range communication, high resistance to interference, and uniform coverage within an area^38^. Table 2 presents the most important simulation parameters in summary.

**Table 2:** Simulation parameters.

Parameter	Value
Simulator	NS-2.35
Environment size	$2000\times 2000\times 300\,\,\text {m}^{3}$
The number of UAVs	20–100
UAV speed (m/s)	10–40
Primary energy of UAVs (J)	1000
Mobility model	3D GM
Carrier frequency (GHz)	2.4
Transport protocol	UDP
Communication radius (m)	250
Mac layer protocol	IEEE 802.11n
Antenna	Omni-directional
Propagation model	Free-space
Traffic model	Constant bit rate (CBR) traffic
CBR rate (Mbps)	2

### Average end-to-end delay

**Figure 4:**
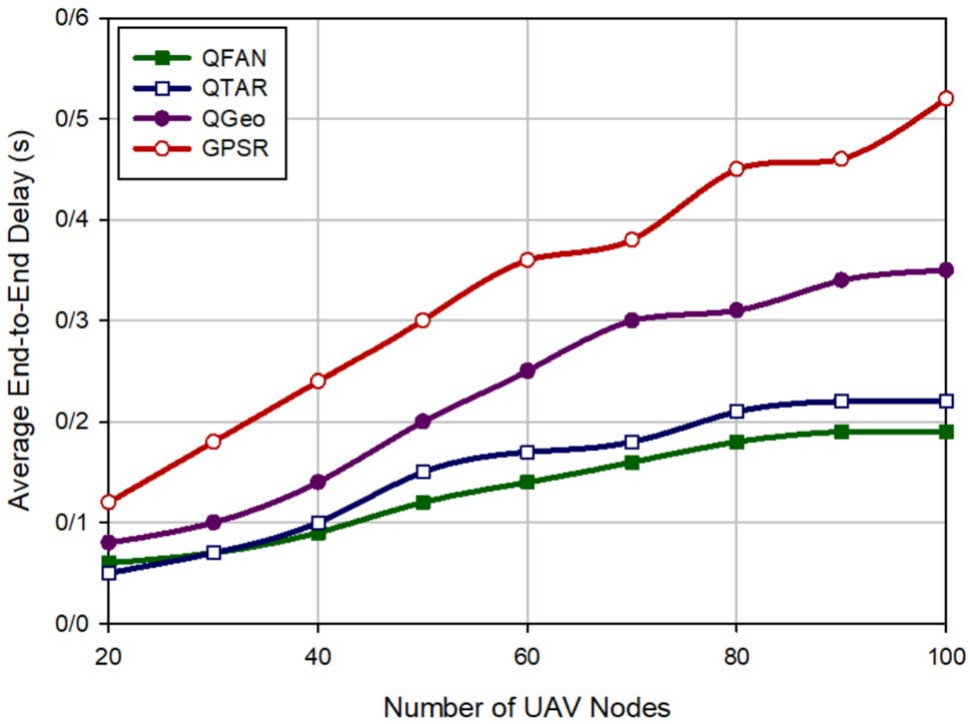
Comparison of end-to-end delay in various approaches in relation to the density of UAVs.

**Figure 5:**
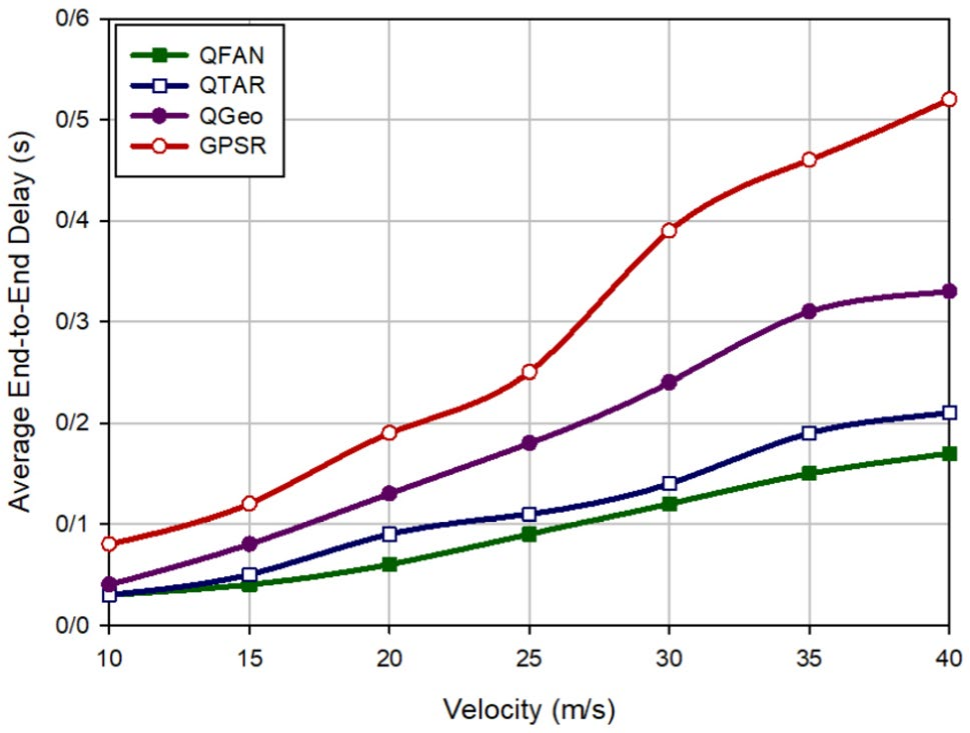
Comparison of end-to-end delay in various approaches in relation to the speed of UAVs.

The average end-to-end delay (Average E2E) is the average required time for successfully transmitting data packets between source and destination nodes. Figure 4 compares the average E2E in various approaches when changing the density of UAVs (between 20 and 100 nodes). Figure 4, shows if there are a small number of UAVs in the network, delay is also low in all routing methods. However, delay will be high when growing the number of UAVs due to the time needed to create paths in the network. Note that our scheme has the minimum delay compared to QTAR, QGeo, and GPSR. QFAN reduces delay by 13.33%, 43.48% and 60.6% compared to QTAR, QGeo, and GPSR, respectively. Moreover, Fig. 5 evaluates delay in different approaches according to speed changes (10–40 m/s). This figure shows the successful performance of our method compared to other schemes. As shown in Fig. 5, QFAN lowers delay by 25%, 52.63% and 68.97% compared to QTAR, QGeo, and GPSR, respectively. According to this figure, we find that delay is increased in all routing approaches when increasing the speed of UAVs in the network because this issue reduces the stability of communication links, which leads to the rapid disconnection of existing routes between nodes and increases the need for finding new paths. This issue increases delay in the network. In general, based on Figs. 4 and 5, it can be found that QFAN has a desirable delay because it considers delay for discovering paths and chooses the paths with less delay for transferring data. In contrast, GPSR focuses only on distance between nodes to choose the next-hop node, this is why it has the worst delay. QTAR considers delay in the routing process and has better performance than QGeo and GPSR. Although, this method has longer delay than QFAN because it requires information of its two-hop neighboring UAVs. On the other hand, QGeo uses spatial information and speed of nodes to choose the next-hop node in the routing operation, but it does not regard delay in this operation. This has increased delay in this method compared with QFAN.

### Packet delivery rate

**Figure 6:**
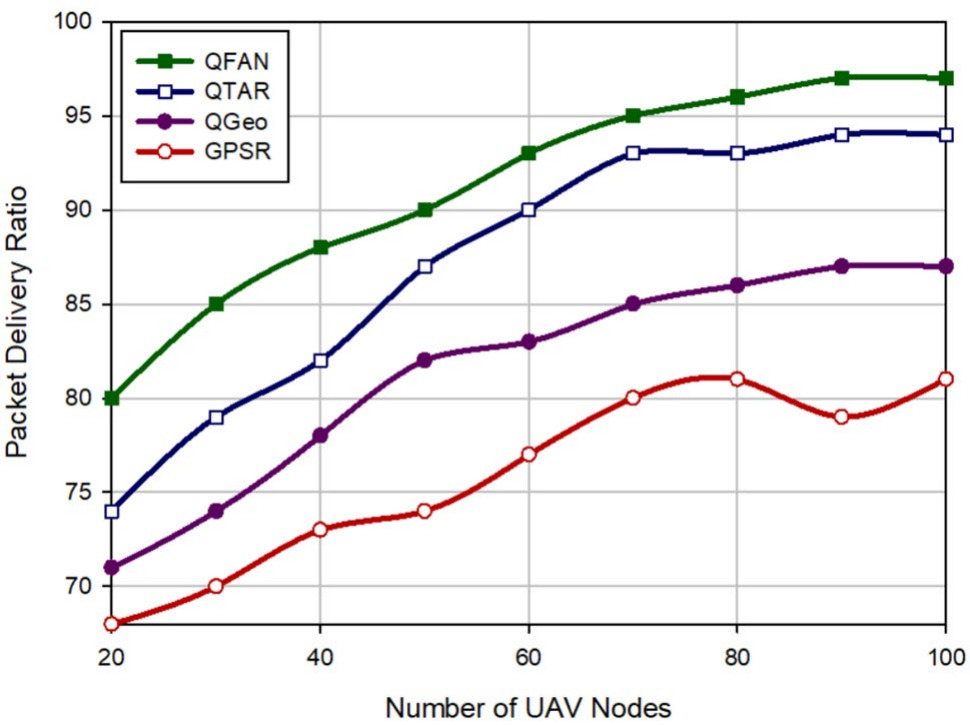
Comparison of packet delivery rate in various approaches in relation to the density of UAVs.

**Figure 7:**
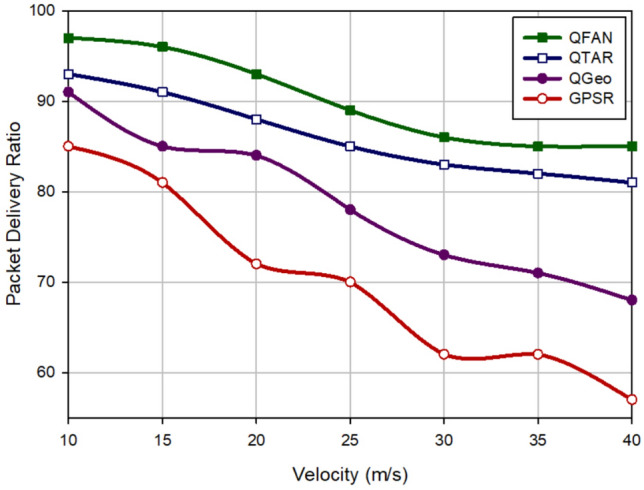
Comparison of packet delivery rate in various approaches with regard to the speed of UAVs.

The packet delivery rate (PDR) is defined as the ratio of packets delivered at the destination to the produced packets. Figure 6 illustrates PDR in various approaches according to the density of UAVs. In accordance with this figure, when growing the number of UAVs, the connections between nodes will be improved. This increases PDR in all routing methods. QFAN has the highest PDR compared to other schemes. It increases PDR by 4.45%, 12.01%, and 20.20% in comparison with QTAR, QGeo, and GPSR, respectively. Figure 7 evaluates various methods in terms of PDR in relation to the speed of nodes. Based on this figure, QFAN has improved this parameter by 4.64%, 14.73%, and 29.03% compared to QTAR, QGeo, and GPSR, respectively. When rising the speed of UAVs, the packet delivery rate decreases in all routing schemes because in this case, the paths made between nodes will be unstable, and the probability of path failure is high. As shown in Figs. 6 and 7, QFAN has a good performance in terms of PDR because it rebuilds the failed paths to lower packet loss due to the path breakage. QFAN considers energy, movement direction, and link quality to form stable paths and reduce path breakages in the network. This will increase PDR in QFAN. In contrast, GPSR and QGEO do not pay attention to energy and link quality. This can increase the probability of path failure, which has lowered PDR in these methods. QTAR has a good performance in terms of PDR. However, its performance is weaker than our method because QFAN uses global information such as the path delay, the path energy, and hop count to calculate the best path, but QTAR depends on local information, such as latency, velocity, and energy of nodes to select the paths.

### Routing overhead

**Figure 8:**
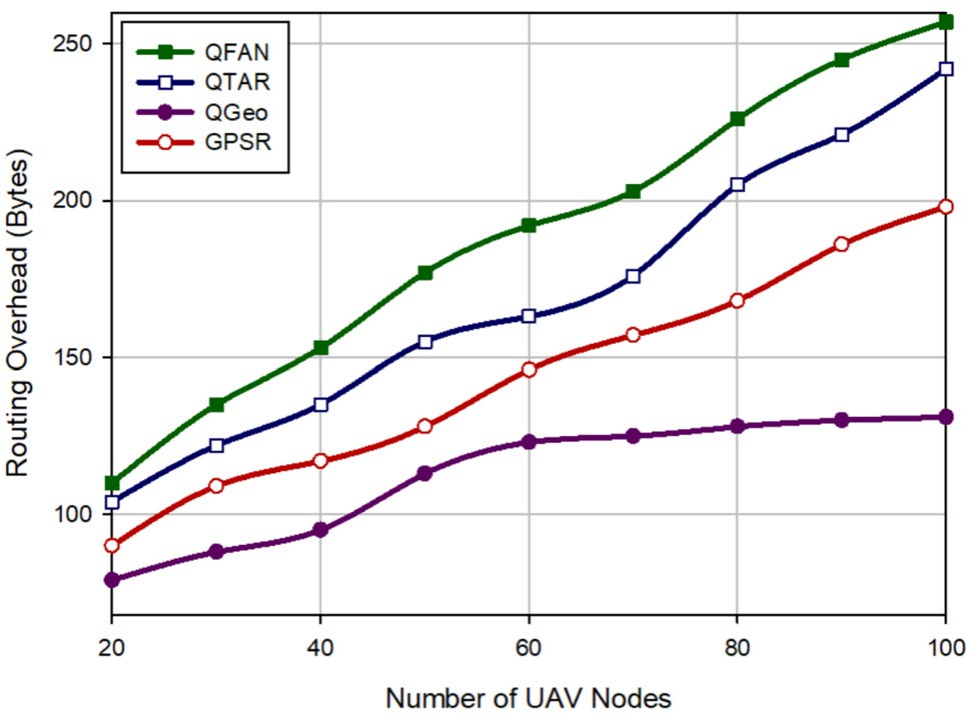
Comparison of routing overhead in various approaches with regard to the density of UAVs.

**Figure 9:**
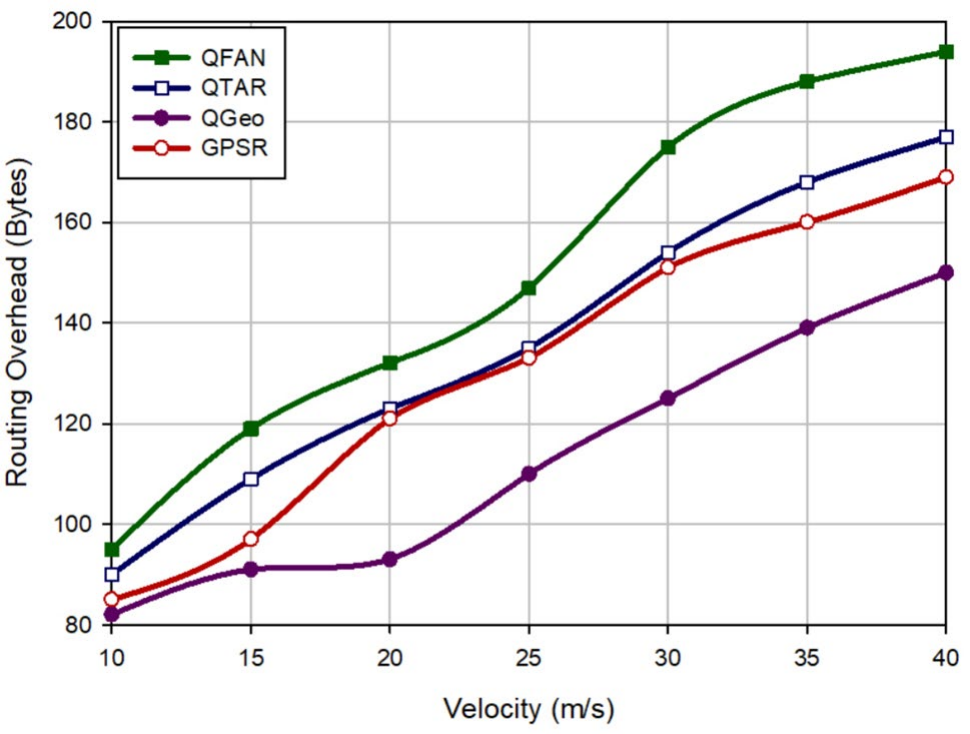
Comparison of routing overhead in various approaches with regard to the speed of UAVs.

Routing overhead means all control packets sent by the routing method. Figure 8 analyzes this parameter in different approaches with regard to the density of UAVs. In this figure, when there are the small number of UAVs, routing overhead is decreased in all routing protocols. When there are the high number of UAVs, they exchange the high number of control messages in the routing operation. According to Fig. 8, QFAN has a higher routing overhead than QTAR (about 11.49%), QGeo (67.79%), and GPSR (30.72%). Figure 9 evaluates different approaches in terms of routing overhead with regard to the speed of UAVs. In accordance with this figure, when the speed of UAVs is increasing in all approaches, routing overhead is high because in this case, the unstable routes are created in the network and the probability of the path failure is very high. As shown in Fig. 9, QFAN has grown routing cost by 9.83%, 32.91%, and 14.66% compared to QTAR, QGeo, and GPSR, respectively. Regarding Figs. 8 and 9, we find that our method has a weaker performance than other methods in terms of routing overhead. This is rooted in the exchange of RREQ and RREP messages when discovering paths in the network. While QTAR and QGeo do not used these control messages in their routing process.

### Energy consumption

**Figure 10:**
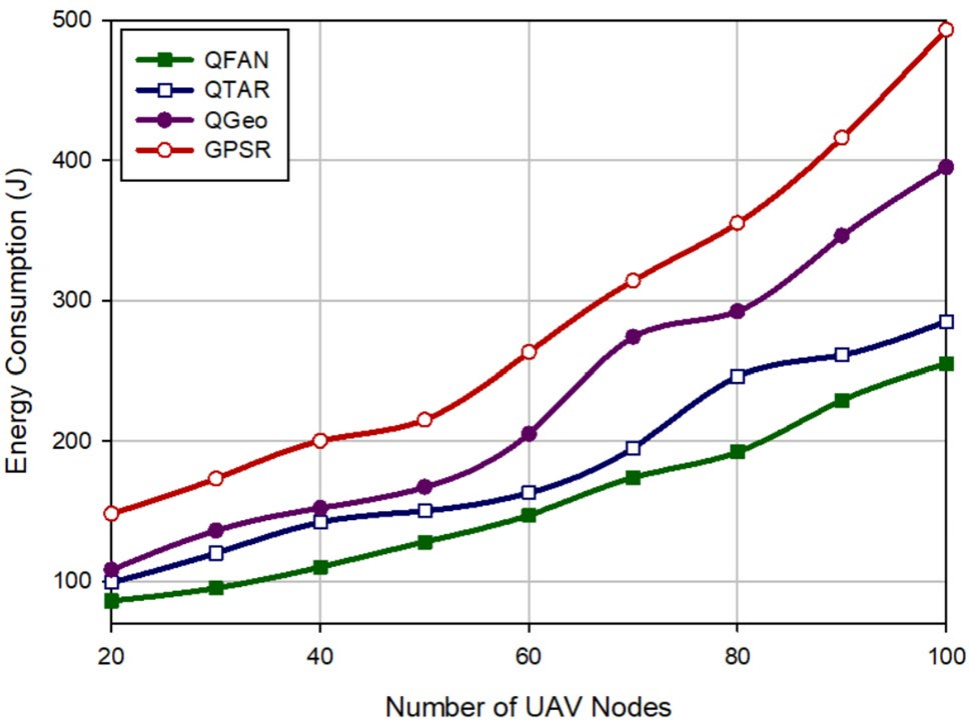
Comparison of energy consumption in various approaches with regard to the density of UAVs.

**Figure 11:**
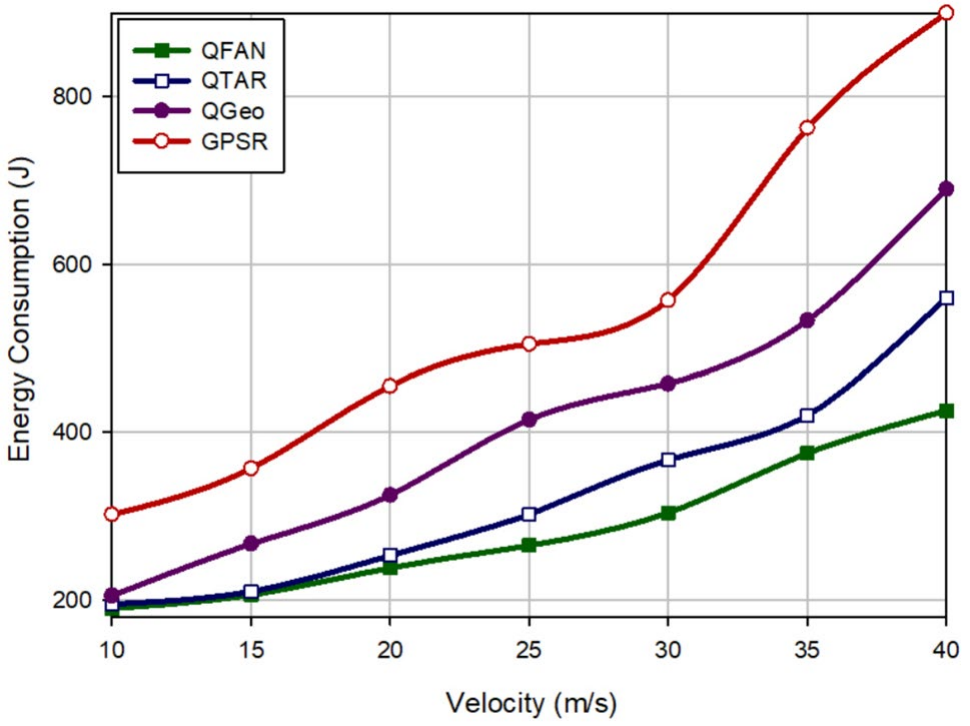
Comparison of energy consumption in various approaches with regard to the speed of UAVs.

This parameter has a great influence on network lifetime and indicates the amount of energy consumed by each UAV when communicating with other nodes. Figure 10 compares energy consumption in different approaches with regard to the density of UAVs. According to Fig. 10, UAVs consume more energy when increasing the density of UAVs because they exchange more control messages for building paths. According to this figure, QFAN has the best performance in terms of energy consumption in comparison with other methods and improves this parameter by 14.75%, 31.76%, and 45.05% compared with QTAR, QGeo, and GPSR, respectively. In addition, Fig. 11 evaluates various methods in terms of energy consumption with regard to the velocity of UAVs. This evaluation shows that QFAN has improved the average energy consumption by 13.14%, 30.73%, and 47.8% compared to QTAR, QGeo, and GPSR, respectively. All methods consume more energy when increasing the speed of UAVs because this issue increases instability of the created paths, which leads to the higher need for rebuilding of failed paths. GPSR and QGeo do not pay attention to the energy of nodes when discovering paths. As a result, they may create low-energy paths, which reduces network lifetime. In contrast, QFAN and QTAR consider energy of UAVs in the routing operation. This balances energy consumption in the network and enhances network lifetime. However, QTAR consumes more energy than QFAN because it needs information of two-hop neighbors. Additionally, QFAN considers different factors such as distance, motion direction, remaining energy, connection quality, number of hops, and delay in the routing operation to build stable paths. Thus, QFAN can reduce path breakages, which improves energy consumption in the network.

### Network lifetime

**Figure 12:**
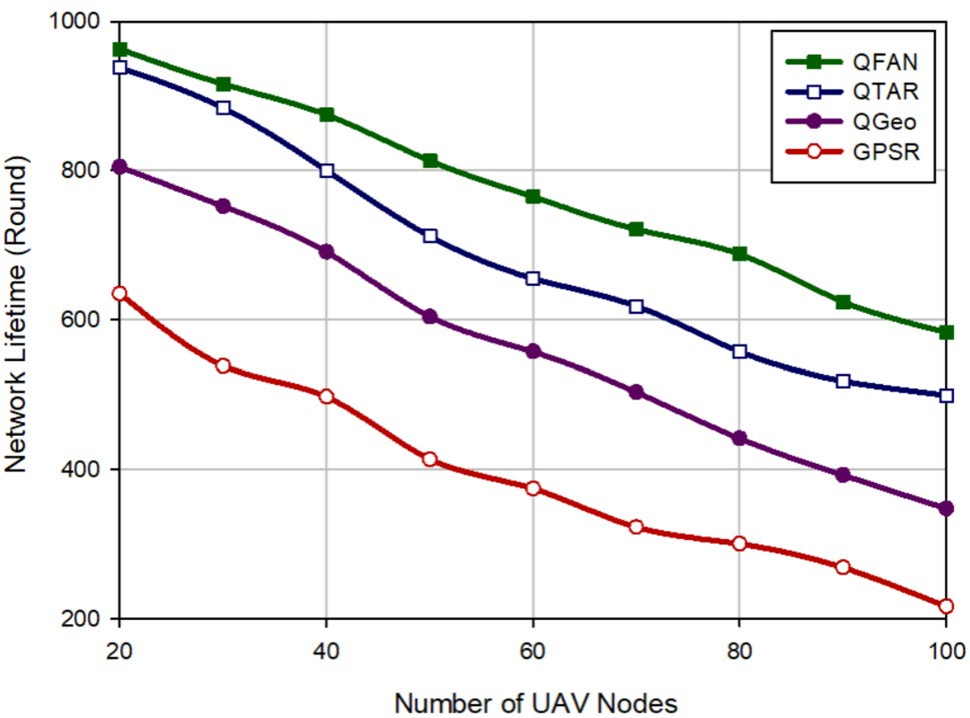
Comparison of network lifetime in various approaches with regard to the density of UAVs.

**Figure 13:**
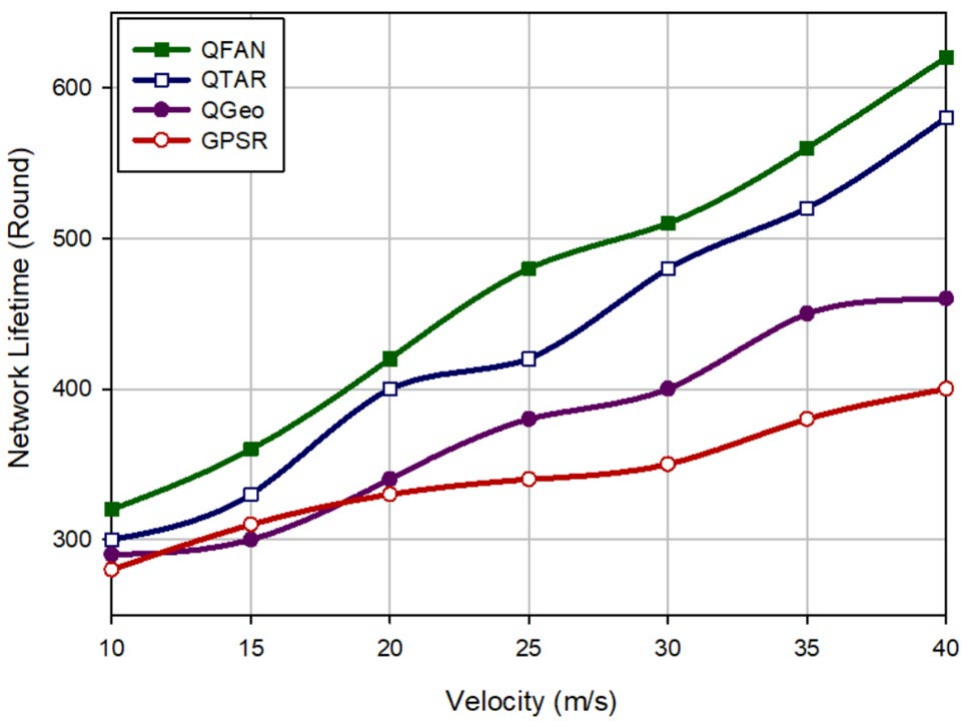
Comparison of network lifetime in various approaches with regard to the speed of UAVs.

The network lifetime indicates the entire routing time until the first node dies (FND) in the network. Figure 12 shows network lifetime in different approaches in relation to the density of UAVs. According to this figure, QFAN has the best network lifetime in comparison with other approaches. It has increased this parameter by 12.41%, 36.45%, and 95% compared to QTAR, QGeo, and GPSR. Figure 13 evaluates various methods in terms of network lifetime with regard to the speed of UAVs. This assessment shows that QFAN has improved this parameter by 7.92%, 24.81% and 36.82% compared to QTAR, QGeo, and GPSR because the proposed method balances energy consumption in the network. As a result, QFAN enhances the network performance in terms of network lifetime. We have described the reasons for this issue in “Energy consumption”.

## Conclusion

In this paper, an energy-aware Q-learning-based routine method (QFAN) was presented for smart air quality monitoring system using FANETs. QFAN involves two parts: route discovery and route maintenance. In the first part, QFAN uses a Q-Learning-based path discovery mechanism to select the best path between source and destination nodes. In this mechanism, each node calculates a filtering parameter for each neighboring UAV. The node chooses a set of licensed neighbors based on this parameter and restricts the search space to quickly achieve the final response (the best path). In the route maintenance phase, QFAN firstly tries to discover and correct paths close to failure. In the second stage, QFAN quickly identifies and replaces the failed paths. Finally, QFAN was simulated by NS2 and compared with QTAR, QGeo, and GPSR in terms of end-to-end delay, packet delivery rate, energy consumption, network lifetime, and routing overhead. We have analyzed the simulation results with regard to density of UAVs and speed changes. The simulation results based on the density of nodes show that our scheme decreases delay compared to QTAR (13.33%), QGeo (43.48%), and GPSR (60.6%). Additionally, QFAN has higher PDR than QTAR (4.45%), QGeo (12.01%), and GPSR (20.20%). Furthermore, our scheme has the minimum energy consumption in comparison with QTAR (14.75%), QGeo (31.76%), and GPSR (45.05%). Moreover, it has the best network lifetime compared to QTAR (12.41%), QGeo (36.45%), and GPSR (95%). However, the routing overhead has been increased slightly in QFAN compared with QTAR (about 11.49%), QGeo (67.79%), and GPSR (30.72%). Also, the simulation results based on speed of nodes show that QFAN has less delay than QTAR (25%), QGeo (52.63%), and GPSR (68.97%). Moreover, it has higher PDR than QTAR (4.64%), QGeo (14.73%), and GPSR (29.03%). In addition, QFAN has less energy consumption than QTAR (13.14%), QGeo (30.73%), and GPSR (47.8%). Moreover, it has higher network lifetime than QTAR (7.92%), QGeo (24.81% ), and GPSR (36.82%). However, QFAN has higher routing overhead QTAR (9.83%), QGeo (32.91%), and GPSR (14.66%). In the future research directions, we seek to focus on multi-path routing methods in FANETs to enhance fault tolerance. Also, we try to apply other artificial intelligence (AI) techniques to provide efficient routing methods in FANETs.

## Data Availability

All data generated or analyzed during this study are included in this published article.
